# Changes in US long-term care facility antibiotic prescribing, 2013–2021

**DOI:** 10.1017/ash.2023.385

**Published:** 2023-09-29

**Authors:** Katryna Gouin, Stephen Creasy, Mary Beckerson, Marti Wdowicki, Lauri Hicks, Sarah Kabbani

## Abstract

**Background:** Antibiotic use (AU) data are needed to improve prescribing in long-term care facilities (LTCFs). CMS requires AU tracking in LTCFs (effective 2017). Although most LTCFs have limited resources for AU tracking, LTCFs contract with LTCF pharmacies to dispense, monitor, and review medications. The objective of our analysis was to report LTCF antibiotic prescribing and characterize temporal changes from 2013 to 2021. **Methods:** We estimated annual systemic AU rates using prescription dispenses and resident census data from PharMerica, a LTCF-pharmacy services provider that covers ~20% of LTCFs nationwide, although the number of LTCFs and residents serviced by PharMerica varied over time (Fig. 1). We included LTCFs with ≥4 months of antibiotic dispensing and 12 months of census data. We identified courses by collapsing the same drug dispensed to the same resident within 3 days of the preceding end date. Course duration was calculated as the difference between the end and dispense dates. We reported yearly AU rates as courses per 1,000 residents and days of therapy (DOT) per 1,000 resident days from 2013 to 2021. We compared AU rates (percentage change) and antibiotic courses by class and agent (absolute percent difference) between 2013 and 2021. **Results:** From 2013 to 2021, AU course rates reported as antibiotic courses per 1,000 residents decreased (percentage change, −28%), with a notable increase in 2020 (Fig. 1). However, the median course duration remained the same (Table 1). The AU decline was mostly driven by decreases in fluoroquinolone courses (absolute difference, −10%, most commonly levofloxacin) and macrolide courses (−2%, most commonly azithromycin) (Figs. 2 and 3). Increases in cephalosporin courses (absolute difference, +7%, most commonly cephalexin) and tetracycline courses (+5%, most commonly doxycycline) were also observed (Figs. 2 and 3). During this period, AU DOT rates reported as DOT per 1,000 resident days decreased (percentage change, −13%) (Table 1). **Conclusions:** The LTCF AU rates, especially for fluoroquinolones, have decreased in recent years with associated shifts in the distribution of antibiotic classes. This finding may be due to CMS stewardship requirements and increased awareness of adverse events, including the FDA fluoroquinolone warnings. The observed increase in 2020 could be secondary to changes in prescribing practices and resident population during the COVID-19 pandemic. Opportunities to improve prescribing in LTCFs include optimizing treatment duration and leveraging LTCF-pharmacy resources to provide stewardship expertise and support AU tracking and reporting.

**Figure:**
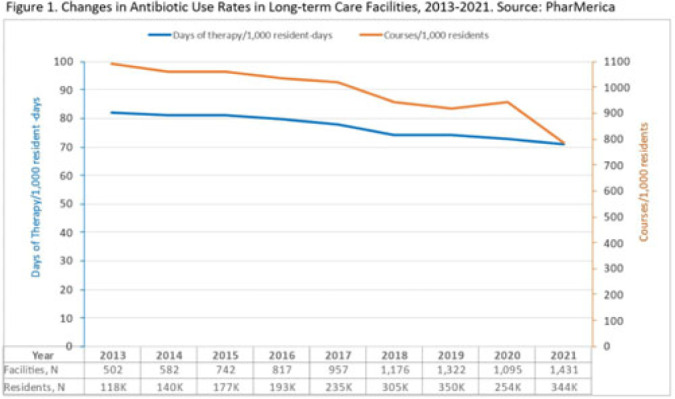

**Figure:**
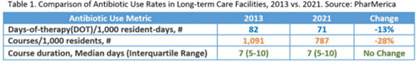

**Figure:**
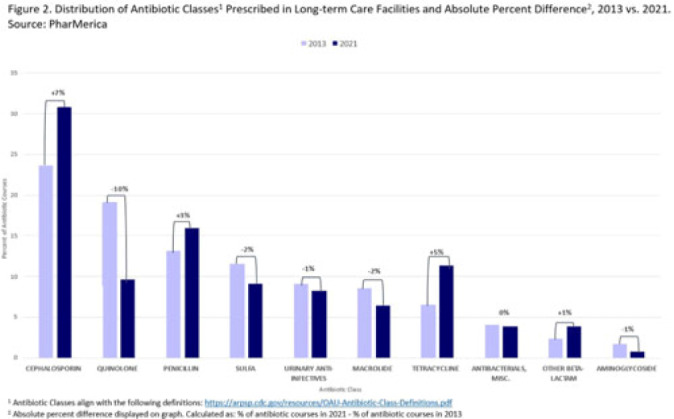

**Figure:**
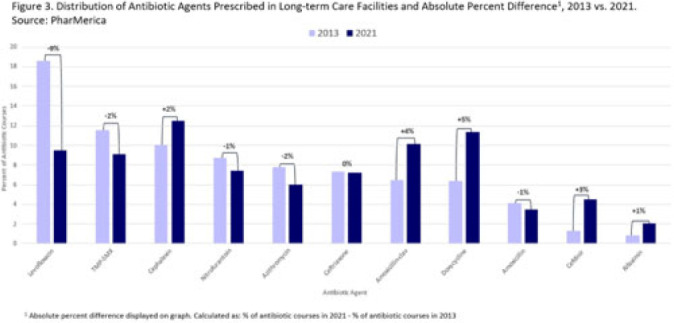

**Disclosures:** None

